# Purinergic and muscarinic modulation of ATP release from the urothelium and its paracrine actions

**DOI:** 10.1152/ajprenal.00291.2013

**Published:** 2013-11-27

**Authors:** Guiping Sui, Chris H. Fry, Bruce Montgomery, Max Roberts, Rui Wu, Changhao Wu

**Affiliations:** ^1^Division of Biochemistry and Physiology, Faculty of Health and Medical Sciences, University of Surrey, Guildford, Surrey, United Kingdom;; ^2^Department of Urology, Frimley Park Hospital National Health Service Foundation Trust, Frimley, Surrey, United Kingdom; and; ^3^School of Medicine, University of Birmingham, Birmingham, United Kingdom

**Keywords:** Urothelium, ATP release, paracrine effect, purinergic, muscarinic, neurotransmitters, sensory

## Abstract

The urothelium is a newly recognized sensory structure that detects bladder fullness. Pivotal to this sensory role is the release of ATP from the urothelium. However, the routes for urothelial ATP release, its modulation by receptor-mediated pathways, and the autocrine/paracrine role of ATP are poorly understood, especially in native tissue. We examined the action of key neurotransmitters: purinergic and muscarinic agonists on ATP release and its paracrine effect. Guinea pig and human urothelial mucosa were mounted in a perfusion trough; superfusate ATP was measured using a luciferin-luciferase assay, and tissue contractions were recorded with a tension transducer. Intracellular Ca^2+^ was measured in isolated urothelial cells with fura-2. The P2Y agonist UTP but not the P2X agonist α,β-methylene-ATP generated ATP release. The muscarinic agonist carbachol and the M_2_-preferential agonist oxotremorine also generated ATP release, which was antagonized by the M_2_-specific agent methoctramine. Agonist-evoked ATP release was accompanied by mucosal contractions. Urothelial ATP release was differentially mediated by intracellular Ca^2+^ release, cAMP, exocytosis, or connexins. Urothelium-attached smooth muscle exhibited spontaneous contractions that were augmented by subthreshold concentrations of carbachol, which had little direct effect on smooth muscle. This activity was attenuated by desensitizing P2X receptors on smooth muscle. Urothelial ATP release was increased in aging bladders. Purinergic and muscarinic agents produced similar effects in human urothelial tissue. This is the first demonstration of specific modulation of urothelial ATP release in native tissue by purinergic and muscarinic neurotransmitters via distinct mechanisms. Released ATP produces paracrine effects on underlying tissues. This process is altered during aging and has relevance to human bladder pathologies.

the role of the urothelium as a novel sensory structure has generated intense interest in recent years and may serve similar sensory and motor functions in other mucosa-lined visceral organs. The key evidence for an active role of the urothelium is that distension of the urinary bladder resulted in the release of neurotransmitters from the urothelium, including ATP and ACh (18, 54). The subsequent identification of P2X3 receptors on suburothelial nerves, as well as the attenuation of afferent activity and urinary retention in mice lacking P2X3 receptors (14, 50), emphasized the importance of the urothelium as a structure that may specifically influence afferent activation and bladder function (4, 6, 42). Changes to urothelial receptor expression (3, 7, 9, 43) and ATP release (5, 27, 48) in tissues or cultured cells obtained from overactive bladders further demonstrated the pathological implications of this tissue, which may lead to specific drug targets for the management of overactive bladders. However, the functional pathways mediated by the various urothelial receptors (3, 7, 10, 12, 41) remain poorly understood. In addition, drugs currently used to manage the overactive bladder work better in the filling phase rather than the voiding phase of the micturition cycle (2) when the detrusor muscle is relaxed. Thus, these drugs may influence a sensory pathway associated with bladder filling rather than the voiding phase of the micturition cycle when motor nerves contract detrusor smooth muscle.

Central to urothelium-mediated responses is the release of ATP, the primary mediator that senses bladder distension and the associated purinergic activation as a result of that release. However, the routes for ATP release and its control are poorly understood, especially in native tissue. Of further interest are other relevant in situ tissue properties of the urothelium that could underlie urothelial ATP release and sensory activation. Furthermore, the urothelium is in close contact with a suburothelium, which, together, is called the mucosa and may be considered as a functional entity. The suburothelium contains a functional syncitium of interstitial cells (46, 47) that respond to ATP to produce excitatory responses and a dense network of blood vessels and afferent nerve fibers.

The functional relevance of urothelial ATP release has never been experimentally demonstrated. ATP released from the urothelium may have an autocrine function or may act in a local paracrine way to influence afferent nerve, interstitial cell, or detrusor smooth muscle function. This study aimed to *1*) characterize ATP release and its modulation by purinergic and muscarinic receptor agonists, the major excitatory neurotransmitters, in intact guinea pig urothelial tissue and *2*) explore its paracrine effect on smooth muscle function. Further aims were to determine if these processes were altered in the aging bladder and whether similar phenomena were present in the human bladder.

## MATERIALS AND METHODS

### Animal and Human Tissue Preparations

Young (2–3 mo) and old (16–18 mo) male Dunkin-Hartley guinea pigs were used. At 2–3 mo old, guinea pigs are capable of breeding but have not yet reached full maturity. Guinea pigs at 16–18 mo old have been shown to have age-related structural or functional changes in the body, including the bladder, and represent the late middle age/elderly transition (early old age) but are not senescent (19, 21, 23, 24, 58). Animals were humanely euthanized by a schedule 1 procedure in compliance with the United Kingdom (UK) Animals (Scientific Procedures) Act of 1986. This study was approved by the local Ethics Committee and followed UK Home Office regulations. The urinary bladder was quickly removed and placed in a HEPES-buffered Ca^2+^-free solution.

Guinea pig bladders were cut from the neck to the dome and laid out as a sheet with the luminal face uppermost. The mucosa (urothelium and suburothelium) was carefully separated from the underlying detrusor layer by microdissection along the natural cleavage plane with microdissection scissors under a dissection microscope; any attached tissue was cleared away. Longitudinal mucosal sheets (urothelial sheets) were cut and mounted in a customized horizontal perfusion trough made from a perspex block with a water-jacketed system to maintain the temperature at 37°C. The tissue trough had dimensions of 4 mm (width) × 5 mm (depth) × 30 mm (length). The specimen was tied by suture thread (size 7/0) at one end to a fixed hook and at the other end to a micromanipulator lever to avoid accidental stretching, which would cause ATP release. The tissue was mounted under microscope vision to avoid any accidental damage. The preparation was submerged in physiological saline solution to measure ATP release or contractile activities. After being mounted, tissue was perfused for at least 1 h before experimentation. In a subset of experiments, one end of the sheet was tied up to an isometric tension transducer to record contractions of the preparation.

The dissected mucosa was also used to prepare single urothelial cells by enzymatic dissociation as previously described (47). Briefly, the urothelial sheet was incubated in collagenase-containing HEPES-buffered Ca^2+^-free solution (see *Solutions*) at room temperature for 10 min, cut into small pieces, and then gently stirred at 37°C for a further 10 min. After incubation, the enzyme solution was discarded by centrifugation (700 *g*), and the cell pellet resuspended in HEPES-buffered Ca^2+^-free solution and stored at 4°C for use on the same day.

Urothelium-attached detrusor muscle strips were also isolated from the bladders and attached to an isometric tension transducer for contractile experiments (see *Measurement of Isometric Tension*).

For a set of specific control experiments, the mucosa was further separated into epithelial and submucosal layers by carefully peeling the epithelium layer off the mucosa with fine-tipped forceps under microscopic guidance.

Human bladder mucosa biopsies were obtained by cystoscopy from male patients (59–80 yr old) undergoing diagnostic endoscopy. Full Research Ethics Committee approval and informed patient consent (in accordance with the Helsinki Declaration) were obtained. Human specimens were treated similarly to guinea pig tissue. The mucosa was tied up in the perfusion trough for experiments to measure ATP release and contractile activity as detailed below in *Measurement of ATP Release* and *Measurement of Isometric Tension*.

All preparations were superfused in HEPES-buffered physiological saline solution at pH 7.4 and 37°C.

### Solutions

The nominally Ca^2+^-free HEPES-buffered Tyrode's solution used for tissue isolation and storage contained (in mM) 132 NaCl, 4.0 KCl, 1.0 MgCl_2_, 0.4 NaH_2_PO_4_, 10 HEPES, 6.1 glucose, and 5.0 Na pyruvate (pH 7.4). The enzymes added to the solution for cell dissociation were collagenase type I (1.0 mg/ml, Worthington Biochemical, Lakewood, NJ), hyaluronidase type I-S (0.25 mg/ml) and type III (0.25 mg/ml), trypsin inhibitor type II-S (0.45 mg/ml) and BSA (2.5 mg/ml). For experiments, the normal HEPES-Tyrode's solution also contained 1.8 mM CaCl_2_ with an osmolarity of 311.2 mosM. Hypotonic HEPES-Tyrode's solution (70% osmotic strength, osmolarity: 217.8 mosM) was diluted with deionized water. Na_2_ATP, carbachol, Na_2_UTP, α,β-methylene ATP (ABMA), oxotremorine, atropine, methoctramine, and 4-diphenylacetoxy-*N*-methylpiperidine were made as aqueous stock solutions at least 1,000 times the final concentrations used in experiments. Sigma ATP assay mix was used for measurements of ATP. All chemicals were from Sigma unless otherwise indicated. The concentrations used for agonists were those that generated a reproducible effect but would not cause significant desensitization and were above EC_50_ but below the maximal effect. The concentrations for antagonists were based on their affinity for the receptors (subtypes) and the agonist concentrations used.

### Measurement of ATP Release

Mucosa preparations were incubated and superfused longitudinally in the perfusion trough with HEPES-buffered Tyrode's solution at a constant flow rate of 2 ml/min. The superfusate in the vicinity of the mucosa, at a fixed point two-thirds downstream along the length of the tissue (∼1–2 mm away and at the same level as the tissue), was sampled with a micropipette tip and snap frozen for later ATP assay. The sampling volume was 30 μl to minimize the dilution effect and mechanical disturbance. The agonists were delivered to the urothelial preparation through the superfusate. The first sampling was taken 1 min after drug infusion, and subsequent samples were obtained at an interval of 2 min for the first 8–10 min and thereafter at 5-min intervals for longer observations. Peak steady-state values were taken as drug effects. Mean values before and after a drug intervention were taken as control (baseline) values. In stretch experiments, the urothelial mucosa was mechanically stretched (within 2 s) to 120% of the original length for 2 min before the tissue was relaxed; the superfusate was then sampled at 30-s intervals for the first 2 min and thereafter at 2-min intervals for a further 10 min. Peak steady-state values after the stretch were taken as stretch effects against the baseline before and after the stretch. The quantity of ATP released into the superfusate was determined directly using a firefly luciferin-luciferase assay where the emitted light was proportional to the concentrations of ATP. The complete Sigma ATP assay mix (FLAAM, Sigma) was diluted with the supplied assay buffer as per the manufacturer's instructions. Luminescence intensity was read using a luminometer (Glomax 20/20, Promega) and calibrated with an ATP standard on the day of each experiment with luminescence as a linear function of concentration on a calibration log-log plot over the range of 100 pM to 1 μM. Appropriate controls were carried out in background solutions using the solvents and chemicals tested. The detection limit of the system was 5–10 fmol ATP. Note that the estimated quantity of released ATP is likely to be an underestimate of the amount released into the microenvironment of the tissue due to the diffusion barrier, tissue ecto-ATPase activity, and flow. However, this nonrecirculating and nonstatic experimental setup offers constant flow to remove the accumulated metabolites and allows dynamic changes to be adequately captured. It also permits steady delivery of a specific chemical intervention through perfusion and reliable quantification of its effect against the control.

### Measurement of Lactate Dehydrogenase Activity

To exclude the possibility of ATP release due to potential tissue damage, the integrity of the urothelium was assessed by measuring the activity of lactate dehydrogenase (LDH) released to the perfusate, sampled as described above. The complete Sigma LDH activity assay kit was used with LDH as the standard.

### Measurement of Intracellular Ca^2+^

Intracellular Ca^2+^ concentrations were measured by epifluorescence microscopy as previously described (45). Urothelial cells were incubated with the Ca^2+^-sensitive fluorochrome fura-2 AM (5 μM, Merck) at 25°C for 20–30 min. For experimentation, cells were continuously superfused with HEPES-buffered Tyrode's solution in a perfusion chamber on an inverted microscope stage. The object cell was illuminated alternately at 340 or 380 nm at 50 Hz; the fluorescent light intensity was measured between 410 and 510 nm. The ratio of fluorescent light intensity when illuminated at 340 and 380 nm was used as an index of intracellular Ca^2+^ concentration and calibrated using an in vitro method in the presence of varying concentrations of free Ca^2+^ (52).

### Measurement of Isometric Tension

Human and guinea pig bladder mucosal sheets or urothelium-attached detrusor muscle strips were mounted in the perfusion trough and tied to an isometric tension transducer and bridge amplifier. The output was sampled at 10 Hz, filtered at 5 Hz, digitized, and stored on a PC for offline analysis. Tissue preparations were continuously superfused in HEPES-buffered Tyrode's solution, and the drug interventions were delivered via the superfusate as described above.

### Data Analysis

Data are expressed as means ± SE; *n* is the number of animals or patients unless otherwise indicated. Student's *t*-tests examined two paired and unpaired normally distributed data sets, and nonparametric equivalent tests were used for data sets of unknown distribution. ANOVA and a Bonferroni post hoc test were used for multiple comparisons; correlation analysis was performed for the association between two variables. The significance of an association between two variables was estimated from a calculation of the Pearson correlation coefficient (*r*) and the subsequent calculation of *t* by the following relationship: $t=r\sqrt{(n-2)/\left(1-r^{2}\right)}$ and a final estimate of *P*, where *n* is the number of data points. Raw data were processed and analyzed with Clampfit software. The null hypothesis was rejected at *P* < 0.05.

## RESULTS

### Release of ATP From Mucosa Sheets and Modulation by Neurotransmitters

#### Intrinsic ATP release in mucosa sheets.

**Fig. 1.**
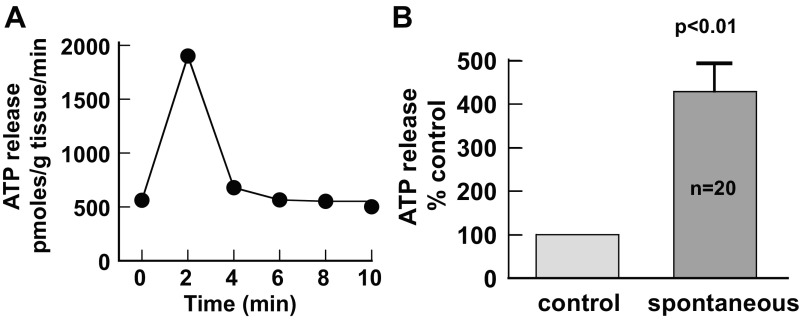
Spontaneous surges of ATP release in guinea pig urothelial tissue. *A*: record of ATP release showing a spontaneous increase in urothelial mucosa. *B*: averaged data from 20 bladders. Control values were taken as 100%.

There was a significant release of ATP from intact sheets of guinea pig bladder mucosa in the absence of exogenous stimuli, as shown by a measurable ATP concentration in the superfusate sample (561 ± 80 pmol·g tissue^−1^·min^−1^, *n* = 61 bladders); there was no ATP measured in superfusate samples in the absence of a tissue preparation. In a subset of control experiments, the mucosa was further separated into uroepithelium and suburothelial layers, and total ATP released from each tissue layer was collected and measured in a static system (vial tubes). ATP released from the uroepithelium was 324 ± 43% of that from the suburothelium, and this accounted for 75.5 ± 2.9% release from the mucosa. Thus, the main source of ATP release (>70%) was from the uroepithelium. With 30–40% of the mucosal preparations, transient, spontaneous increases of ATP release occurred (Fig. 1*A*), which were, on average, four times above basal levels (Fig. 1*B*). These spontaneous release activities reached a peak after 2 min with a mean value of 2.48 ± 0.19 min (means ± SE, *n* = 63 events). The active release of ATP from the intact tissue is consistent with the concept that the mucosal sheet forms a cellular network.

#### Modulation of mucosal ATP release by neurotransmitters.

**Fig. 2.**
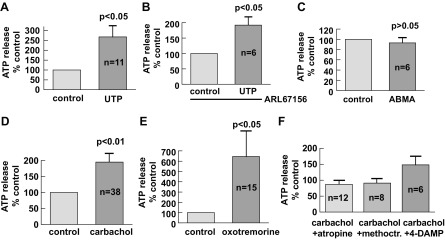
Effect of purinergic agonists and muscarinic agents on ATP release from guinea pig urothelial mucosa. *A*: the P2Y agonist UTP (100 μM) increased ATP release. *n* = 11 bladders. *B*: the UTP-augmented ATP release persisted in the presence of ARL67156 (100 μM). *n* = 6 bladders. *C*: no effect was seen with the P2X agonist α,β-methylene ATP (ABMA; 10 μM). *n* = 6 bladders. *D*: carbachol (50 μM) induced ATP release from the guinea pig urothelium. *E*: oxotremorine (50 μM) generated ATP release. *F*: effect of muscarinic antagonists on carbachol-induced ATP release [atropine (1 μM), methoctramine (100 nM), 4-diphenylacetoxy-*N*-methylpiperidine (4-DAMP; 10 nM)]. The control represents the level of ATP release in the presence of antagonist alone for each intervention and was taken as 100%.

The role of the major bladder neurotransmitters, purinergic and muscarinic receptor modulators, in the regulation of ATP release was investigated. To examine the possibility of ATP-induced ATP release via an autocrine action, we used UTP, a P2Y agonist, instead of ATP to distinguish between P2X and P2Y subtypes and also to minimize the confounding influence of ATP itself on ATP measurements. UTP (100 μM) significantly enhanced ATP release (Fig. 2*A*). The effect of UTP on ATP release could not have been caused by a possible inhibition of ecto-ATPase in the tissue to enhance ATP levels, as blocking of ecto-ATPase activity with ARL67156 did not affect the stimulatory effect of UTP, consistent with a specific action on the receptors (Fig. 2*B*). In contrast, the P2X-selective agent ABMA had no significant effect on ATP release (Fig. 2*C*). These observations suggest an autocrine and/or paracrine effect of ATP to enhance further ATP release in native urothelial mucosa (see discussion) mediated via P2Y receptors.

The muscarinic agonist carbachol (50 μM) produced an increase in urothelial ATP release (Fig. 2*D*). Oxotremorine, a muscarinic agonist with a preference for M_2_ receptors (22, 37), was more effective (Fig. 2*E*). These excitatory effects were diminished in the presence of the general muscarinic antagonist atropine (1 μM, *P* < 0.05 vs. carbachol; Fig. 2*F*). In further experiments, the M_2_-selective antagonist methoctramine [100 nM, a concentration generating an M_2_-selective effect (35)] abolished the effect of carbachol on ATP release (*P* < 0.05 vs. carbachol; Fig. 2*F*). However, ATP release in the presence of the M_3_-selective muscarinic receptor antagonist 4-diphenylacetoxy-*N*-methylpiperidine (10 nM) (59) was not significantly different from that in carbachol alone (*P* > 0.05 vs. carbachol; Fig. 2*F*). These data suggest that the M_2_ subtype is involved in muscarinic neurotransmitter-induced ATP release from the bladder mucosa.

#### LDH activity.

Control experiments (*n* = 4) showed that in mucosal preparations where there was significant spontaneous release of ATP or agonist-triggered ATP release, the level of LDH activity in the superfusate sampled as described above was below the detection level (0.02 U/ml) for the assay kit. Thus, it is unlikely that tissue damage could have contributed to the spontaneous or agonist-evoked increase of ATP from these preparations.

### Mechanisms Underlying Mucosal ATP Release

#### Urothelium contraction and its relation with ATP release.

**Fig. 3.**
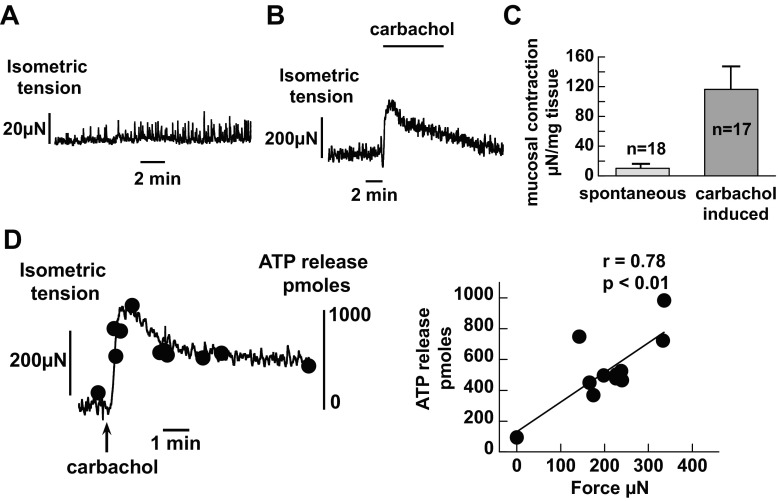
Contractile properties of guinea pig urothelial mucosa. *A*: record of spontaneous contractile activity of a guinea pig bladder mucosal preparation. *B*: carbachol (50 μM) generated mucosal contraction. Note that carbachol-induced contracture on top of the spontaneous contractions. *C*: averaged values of mucosal contractions. *n*, number of bladders. *D*: relationship between carbachol-induced contraction and ATP release in guinea pig urothelial mucosa. *Left*, concomitant mucosal contraction and increase of ATP release (●) induced by carbachol (50 μM). ATP was sampled at various time points before and after carbachol application. *Right*, correlation between the two variables from the experiment.

**Fig. 4.**
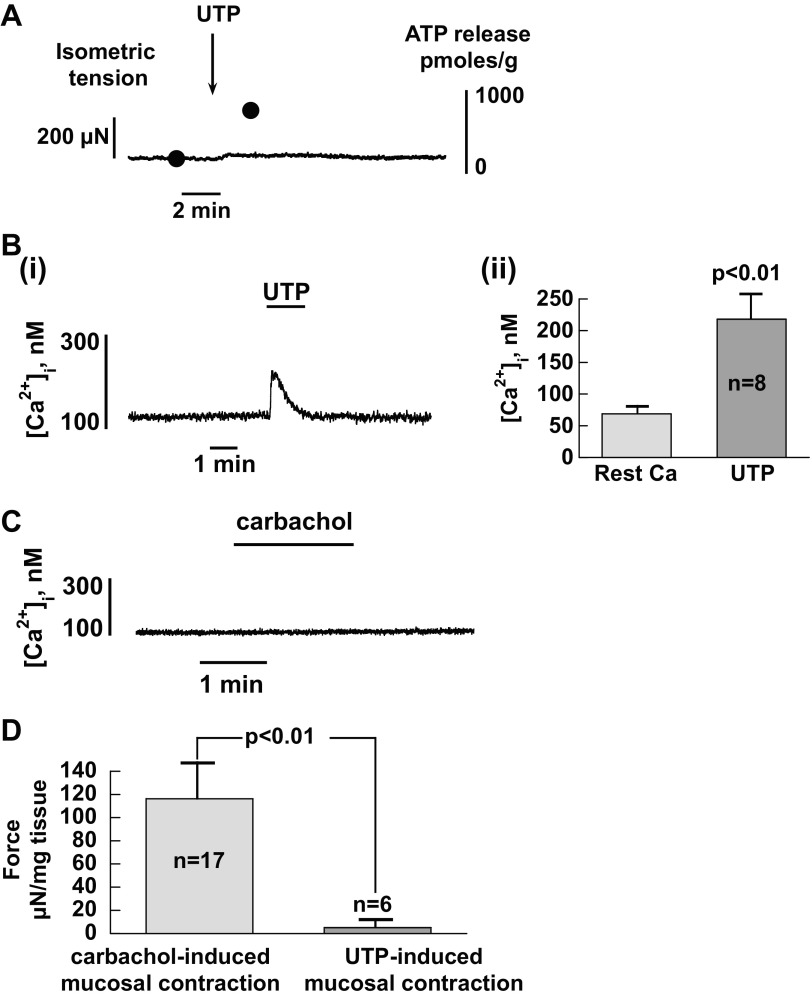
Differential actions of UTP and carbachol on mucosal contraction and intracellular Ca^2+^ concentration ([Ca^2+^]_i_). *A*: changes to urothelial mucosa contraction and ATP release during UTP intervention: UTP (100 μM) generated significant ATP release with only a small effect on contractility, in contrast to carbachol (Fig. 3). *B*: effect of UTP on intracellular Ca^2+^. *i*, UTP (100 μM) generated a Ca^2+^ transient in a urothelial cell; *ii*, averaged data from 8 bladders. *C*: lack of effect on intracellular Ca^2+^ by carbachol (100 μM). *D*: comparison of UTP- and carbachol-induced mucosal contractions. Values are averaged data. *n*, number of bladders. Records are from guinea pig preparations.

Small spontaneous contractions were observed in >60% of urothelial sheets (Fig. 3, *A* and *C*). This intrinsic activity occurred at an average frequency of 0.23 ± 0.04 Hz with an instantaneous frequency of 1.45 ± 0.23 Hz during rapid activity. Contractions were also evoked by the muscarinic agonist in a similar proportion (70%) of urothelial sheets (Fig. 3, *B* and *C*). Carbachol-induced mucosal contraction coincided with the rise of ATP release as demonstrated by sequential sampling (Fig. 3*D*). A consistent relationship between the increase of ATP and mucosal contraction was observed in 16 preparations. In addition, an increase of ATP release could also be observed in urothelial sheets that showed no apparent contractions. These results suggest that mucosal preparations are capable of contracting (see discussion) and that this contraction contributes, in part but not exclusively, to the rise of ATP release by muscarinic activation. Mucosal contractions may also contribute to UTP-induced ATP release but to a much lesser extent. A distinction between purinergic and muscarinic pathways was identified in the data shown in Fig. 4. UTP produced smaller contractions with <5% of carbachol-induced contractions yet produced significant ATP release (Fig. 4, *A* and *D*); hence, purinergic receptors appear to regulate ATP release also through a contraction-independent mechanism. Furthermore, UTP elicited a Ca^2+^ rise in isolated urothelial cells, whereas carbachol produced little change to intracellular Ca^2+^ in these cells (Fig. 4, *B* and *C*). Thus, contraction may mainly contribute to carbachol-induced ATP release in the urothelium, but an intracellular Ca^2+^ rise, which has been shown to contribute to ATP release in cultured smooth muscle cells (25), is more likely to be involved in UTP-evoked ATP release.

#### Release of ATP in response to mechanical stretch.

**Fig. 5.**
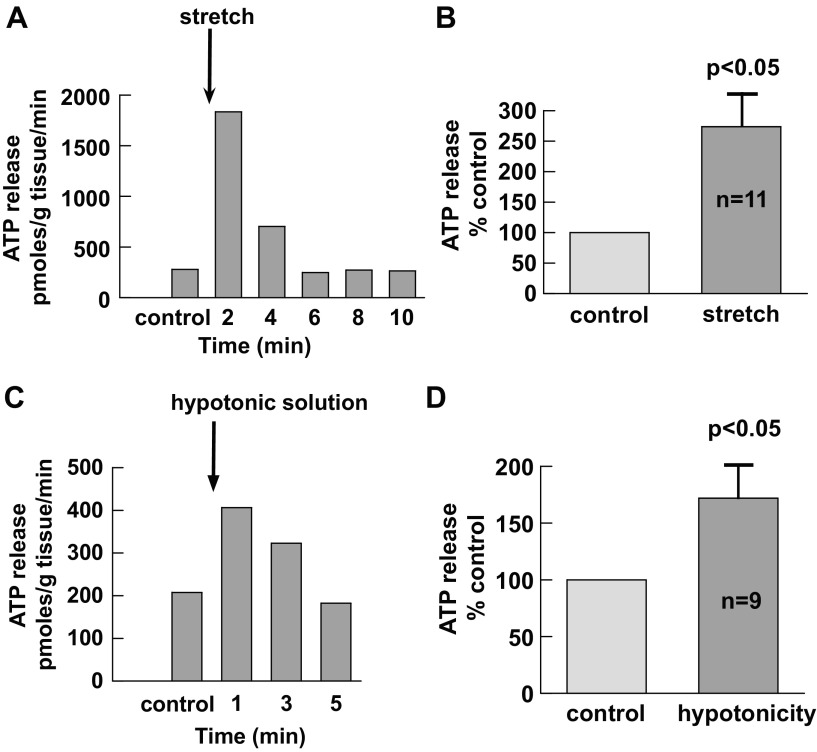
Mechanical stretch-induced ATP release from guinea pig urothelial mucosa. *A*: record of ATP release after stretch (20% increase of original length). *B*: averaged data from 11 bladders. The level of ATP release from the control (without stretch) was taken as 100%. *C* and *D*: effect of hypotonic solution on ATP release in guinea pig urothelial mucosa. *C*: example of urothelial ATP release after challenge with HEPES-buffered Tyrode's solution with tonicity reduced to 70%. *D*: averaged data from 9 bladders. Control values without hypotonic insult were taken as 100%.

One possible mechanism for tissue contraction-induced ATP release is mechanical stretch. The ability of urothelial mucosa to release ATP was thus investigated in response to mechanical stretch. An increase of ATP release from sheets of mucosa was consistently recorded by physical extension of preparations to 120% of the original length (Fig. 5, *A* and *B*). The increase was greatest ∼2 min after the stretch and thereafter diminished. Hypotonic solutions have been used as mechanical stimuli through the generation of cell swelling (5). As shown in Fig. 5, *C* and *D*, hypotonic solutions, with tonicity reduced to 70%, also increased basal ATP release. The responsiveness of urothelial sheets to mechanical stimuli may thus underlie contraction-induced ATP release by agonists.

#### Specific mechanisms underlying intrinsic ATP release.

**Fig. 6.**
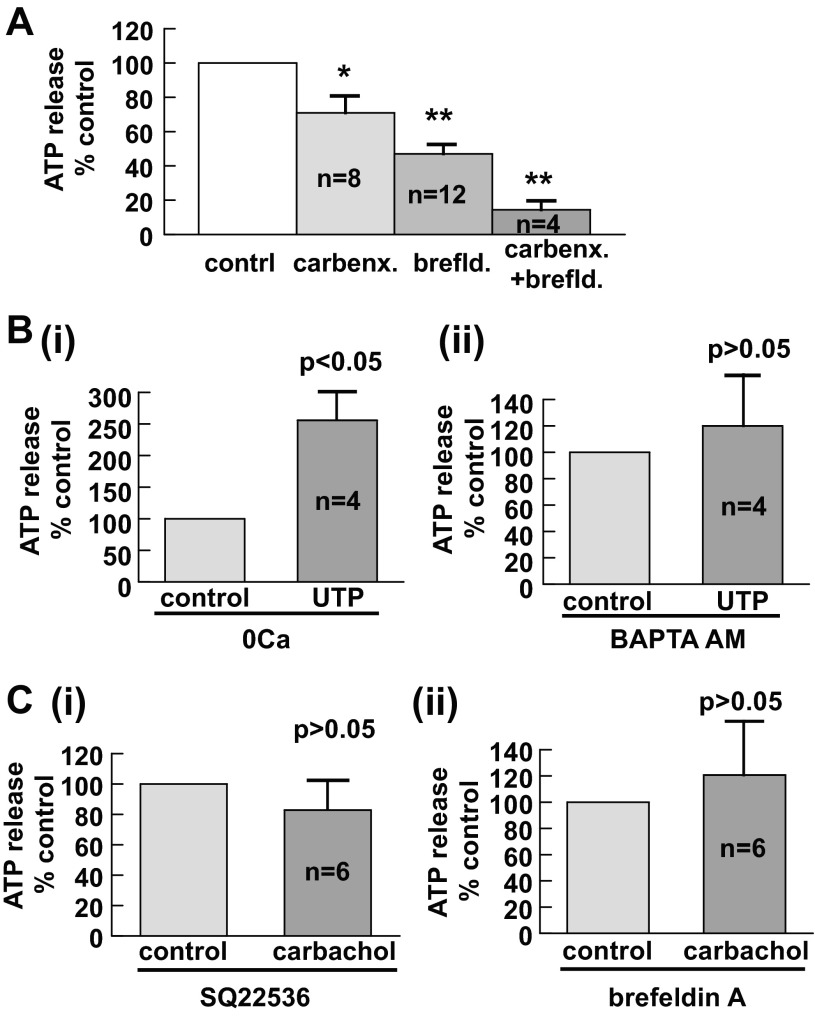
Specific pathways for intrinsic and agonist-evoked ATP release in urothelial mucosa. *A*: conductive and vesicular pathways for intrinsic ATP release. There was a moderate inhibition on intrinsic basal ATP release with the gap junction blocker sodium carbenoxolone (Carbenx; 100 μM), a major suppression of intrinsic ATP release with the vesicular transport inhibitor brefeldin A (Brefld; 10 μM) and an additive effect of both agents. *P < 0.05 and **P < 0.01 vs. control. *B*: Ca^2+^-dependent pathways for UTP-induced ATP release. *i*, Removal of extracellular Ca^2+^ did not suppress UTP-augmented ATP release; *ii*, reducing intracellular Ca^2+^ with BAPTA-AM (10 μM) specifically inhibited UTP-evoked ATP release. *C*: specific pathways for carbachol-induced ATP release. *i*, Absence of the excitatory effect of carbachol on ATP release in the presence of the adenylyl cyclase inhibitor SQ-22536 (100 μM); *ii*, the effect of carbachol was also suppressed by brefeldin A (10 μM).

The possible contributions to intrinsic mucosal ATP release, from the two fundamental pathways, conductive and exocytotic, were further investigated. Blockade of gap junctions with sodium carbenoxolone (100 μM) moderately attenuated the intrinsic ATP release from the urothelium (∼30% reduction; Fig. 6*A*). Inhibition of vesicular release by brefeldin A (10 μM), an agent that disrupts the Golgi complex and vesicle trafficking to the cell surface and hence the fusion of transport vesicles with the plasma membrane (34), produced a major inhibitory effect (>50% reduction) on the intrinsic ATP release (*P* < 0.05, brefeldin A vs. carbenoxolone; Fig. 6*A*). Application of both agents together produced 85.6 ± 5.0% inhibition (*P* < 0.01, *n* = 4), indicating an additive effect. These results indicate that vesicular exocytosis and, to a smaller extent, pennexin conductive fluxes contribute to the majority of intrinsic ATP release from the urothelium.

#### Specific pathways for neurotransmitter-induced ATP release.

The Ca^2+^ dependence of UTP-mediated ATP release from the urothelium was further explored. General removal of extracellular Ca^2+^ by no added CaCl_2_ in the Tyrode's solution plus the addition of the Ca^2+^ chelator EGTA (0.1 mM) had no effect on UTP-induced ATP release (Fig. 6*B*,*i*). However, a selective reduction of intracellular Ca^2+^ with no change in extracellular Ca^2+^ with cell-permeable BAPTA-AM (10 μM) significantly suppressed UTP-induced ATP release (Fig. 6*B*,*ii*). This suggests the importance of Ca^2+^ release from the intracellular store in P2Y receptor-mediated ATP release in this tissue. Both interventions had no effect on basal ATP release (0 Ca^2+^ plus EGTA: 113.3 ± 18.5% of the control, *n* = 6, *P* > 0.05; BAPTA-AM: 109.8 ± 10.1% of control, *n* = 4, *P* > 0.05). The role of vesicular release, which has been shown to mediate basal intrinsic release, was also tested in UTP-generated ATP release. The data showed that in the presence of the vesicular inhibitor brefeldin A (10 μM), UTP still had an excitatory effect on ATP release (179 ± 33% of the control, *n* = 8, *P* < 0.05), indicating that UTP exerts its specific effect on ATP release, which differs from that of intrinsic release.

The Ca^2+^-independent mechanism underlying carbachol-evoked ATP release was also sought. Inhibition of cAMP [by SQ-22536 (100 μM)], a key secondary messenger for M_2_ receptor activation, had no effect on intrinsic ATP release (107 ± 10% of the control, *n* = 6, *P* > 0.05), but the excitatory effect of carbachol was no longer observed (Fig. 6*C*,*i*), consistent with a specific mechanism that requires cAMP. Furthermore, the excitatory effect of carbachol on ATP release was also affected by brefeldin A (10 μM; Fig. 6*C*,*ii*), suggesting an additional involvement of vesicular transport.

### Paracrine Action of Urothelial ATP Release

**Fig. 7.**
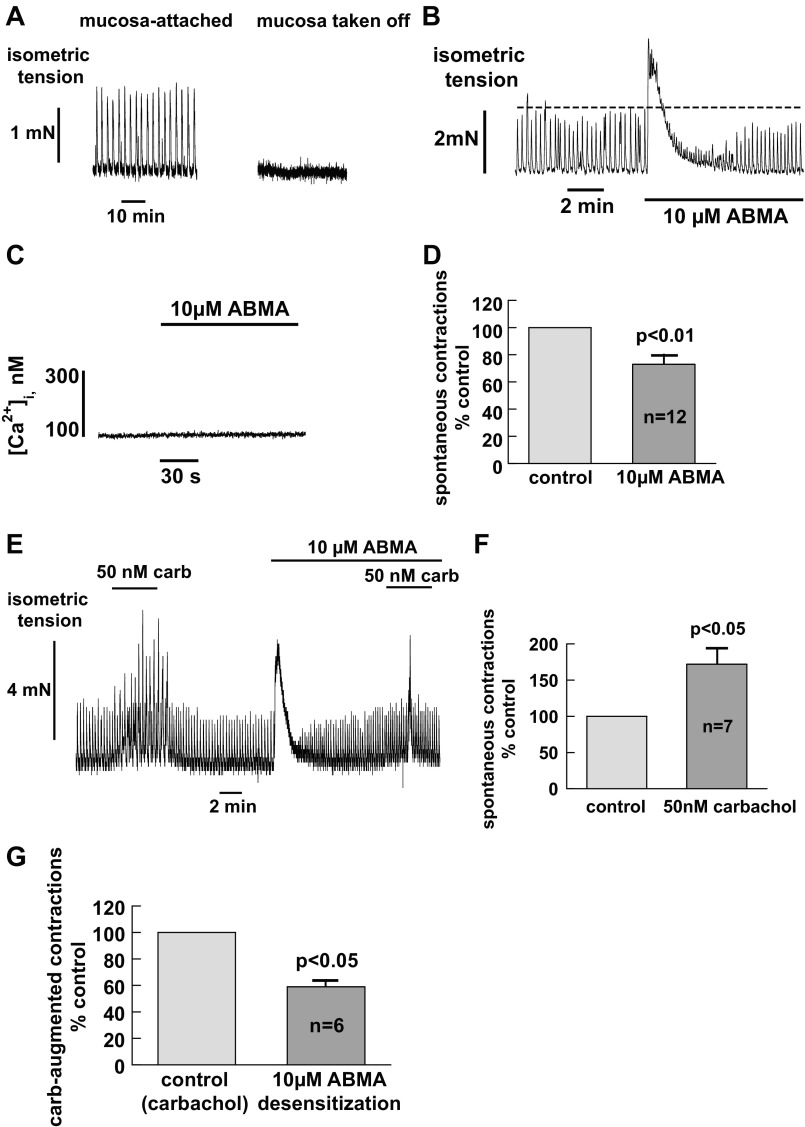
Intrinsic and low concentration carbachol-induced contractile activity in urothelium-intact guinea pig detrusor muscle: involvement of paracrine action. *A*: urothelium dependence of the intrinsic contractile activity of detrusor muscle. Shown is a record of spontaneous activity in a mucosa-intact detrusor muscle strip; the removal of mucosa diminished the activity. *B*: record of the experiment showing the attenuating effect of ABMA on urothelium-dependent spontaneous activity. *C*: there was little effect of AMBA on intracellular Ca^2+^ in a urothelial cell. *D*: averaged data for ABMA intervention on the contractile activity from 12 bladders. *E–G*: augmentation of spontaneous contractions in mucosa-attached detrusor smooth muscle by a subthreshold concentration of carbachol and the effect of ABMA. *E*: record of spontaneous contractions in a urothelium-intact detrusor strip showing an enhanced effect by a low concentration of carbachol and the attenuation of such augmentation after ABMA treatment. *F*: averaged data for the carbachol effect. *n* = 7 bladders. *G*: averaged data for the ABMA effect on carbachol-augmented muscle contractions. *n* = 6 bladders.

Further experiments examined the possible paracrine effect of urothelial ATP release using mucosa-attached detrusor muscle preparations. Spontaneous contractile activity was observed in urothelium-intact muscle preparations, which disappeared when the mucosa was removed (Fig. 7*A*). The tissue remained responsive to stimulation with carbachol after removal of the mucosa, as previously reported (47), which therefore excluded the possibility of tissue damage. As shown in Fig. 7*B*, 10 μM ABMA, a P2X desensitizer, generated a transient contracture, but the spontaneous contraction amplitude was then reduced compared with the control. We interpreted this as an initial direct action of ABMA on detrusor smooth muscle, but as P2X receptors were subsequently desensitized, ATP diffusing from the mucosa generated smaller spontaneous contractions (Fig. 7*D*). ABMA has been shown to exert a significant action on detrusor smooth muscle (51) but no effect on urothelial cells (Fig. 7*C*). This suggests that the mucosa-dependent spontaneous contractions were partly mediated through the activation of P2X receptors on smooth muscle by ATP released from the urothelium, although the net ATP reaching smooth muscle is subject to local regulations (see discussion). In mucosa-attached detrusor muscle, a subthreshold concentration of carbachol (50 nM), which had no direct effect on detrusor muscle, augmented the spontaneous activities, consistent with an effect via muscarinic receptors on mucosa (Fig. 7, *E* and *F*). The low concentration of carbachol was chosen here to demonstrate the clear difference between mucosa-intact muscle and denuded muscle. Carbachol-enhanced muscle activity was also suppressed by prior application of ABMA to desensitize P2X receptors (Fig. 7*E*). This suggests that the carbachol-induced muscle contraction also involves an activation of smooth muscle P2X receptors due to urothelial ATP release. Similar results were observed in five other preparations with a mean reduction to ∼60% of the control (Fig. 7*G*).

### Relevance to Aging and Human Conditions

#### Effect of aging on ATP release.

**Fig. 8.**
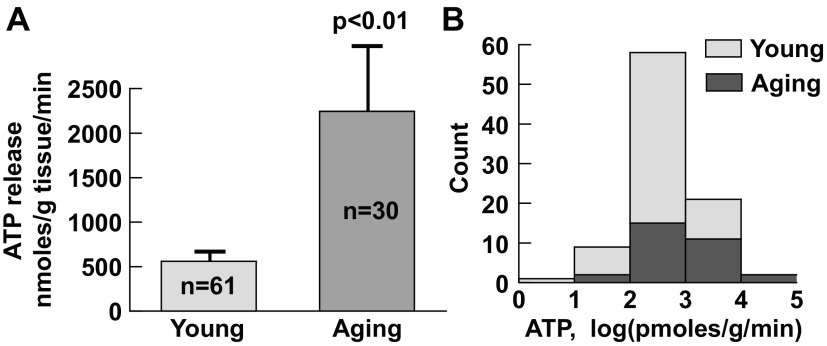
Intrinsic ATP release in urothelial mucosa from guinea pig urinary bladders. *A*: average levels of ATP release in young (2–3 mo) and aging (16–18 mo) bladders. *n*, number of animals. *B*: histogram of ATP release (log-transformed values) from both groups showing the greater proportion of aging bladders at the higher level end of the distribution.

Intrinsic ATP release was also measured in mucosal preparations from aged guinea pigs (16–18 mo old). The level of release in tissue from old guinea pigs was significantly greater than that in tissue from young animals (Fig. 8*A*), suggesting that the process of intrinsic ATP release is altered during aging. Furthermore, ATP release in aging bladders had greater variations (*P* < 0.01 by *F*-test), and more aging bladders released higher levels of ATP than their younger counterparts (Fig. 8*B*).

#### Urothelial ATP release in human preparations.

**Fig. 9.**
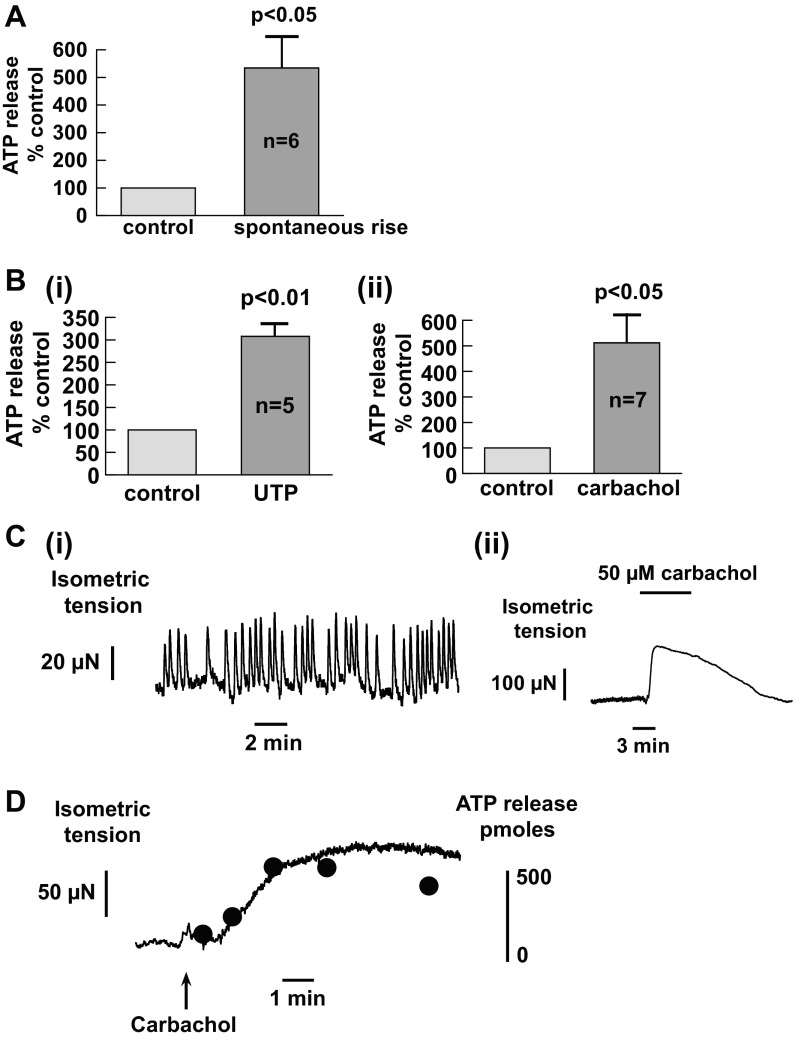
Characteristics of human urothelium preparations. *A*: spontaneous rises of ATP release. Data are from 6 subjects. *B*: neurotransmitter-induced ATP release. *i*, UTP (100 μM)-induced release; *ii*, carbachol (50 μM)-induced ATP release. *n*, number of subjects. *C*: contractile properties of human bladder mucosa. *i*, Spontaneous contractions; *ii*, carbachol (50 μM)-induced response. *D*: mucosal contraction and ATP release. Shown is the time course of contractile activity and ATP release in response to carbachol (50 μM) intervention in a human bladder mucosa.

To ascertain the applicability of the above phenomena to the human bladder, a further series of experiments was performed with human bladder mucosa biopsies with no detrusor muscle attached. Substantial intrinsic ATP release was observed in the human urothelium with a mean value of 369 ± 97 pmol·g^−1^·min^−1^ (*n* = 9 subjects) with spontaneous surges of ATP release (Fig. 9*A*). Both UTP (100 μM) and carbachol (50 μM) evoked significant ATP release in the urothelium (Fig. 9*B*,*i* and *ii*). UTP increased ATP release by an amount similar to that from young guinea pigs (308 ± 34% vs. 268 ± 61% at baseline, *n* = 5 and 11, respectively). However, carbachol-induced release was significantly greater in human preparations than in guinea pig tissue (512 ± 121% vs. 195 ± 23% baseline, *n* = 7 and 38, respectively, *P* < 0.01). Spontaneous contraction and carbachol-induced contractures were also observed in some biopsies (Fig. 9*C*) with an average force of 134 ± 79 μN/mg tissue (*n* = 6 subjects), similar to guinea pig bladder mucosa. These contractions were also accompanied by an increase of ATP release (Fig. 9*D*). All these results demonstrated common functional characteristics and regulatory mechanisms between the animal urothelium and human tissue.

## DISCUSSION

These data show that the native urothelium actively releases ATP and that this process can be modulated by the major neurotransmitters and consequently offers a paracrine mechanism for its release. Moreover, urothelial ATP release is increased in tissue from aging animals. The significance of these observations is supported by the similar characteristics of ATP release in the human urothelium. To our knowledge, this is the first demonstration of specific modulation of ATP release in native urothelium tissue by the major neurotransmitters in a physiological context and with human relevance. These findings may have implications in bladder pathophysiology.

### Intrinsic ATP Release and Effect of Aging

Unstimulated urothelial sheets were shown to be capable of releasing ATP, which generates a purinergic tone to the downstream effectors that express purinoceptors. These include urothelial cells themselves, the suburothelial network of interstitial cells, and afferent nerve endings (17, 31, 50). We also observed significant spontaneous surges of ATP release from the urothelium, which, as suggested by evidence in other tissue types, may have important functional implications (16).

Intrinsic ATP release was significantly upregulated in aging bladders, implying that downstream targets would be stimulated more, and, moreover, the variability of responses was greater, so that the highest levels of ATP release were exclusively observed in these bladders. These changes may reflect the aging process (39) and are consistent with the fact that older individuals are more prone to bladder hyperactivity, a characteristic of detrusor overactivity (11). The mechanisms underlying intrinsic ATP release and the spontaneous surges may be complex, but the spontaneous rises of intracellular Ca^2+^ observed in urothelial cells, in contrast to most other types of epithelial cells, may suggest one underlying mechanism.

### Modulation by Neurotransmitters

We observed significant modulatory effects of purinergic and muscarinic agents on ATP release from the intact urothelial mucosa. Purinergic and muscarinic receptors have been demonstrated in the urothelium by immunocytochemistry, but limited functional experimentation has been carried out, in particular in native tissue. One aim of the study was to determine if receptor ligands controlled ATP release. The ability of the P2Y agonist UTP, rather than the P2X agonist ABMA, to evoke ATP release supports a role for P2Y receptors in this process. UTP instead of ATP as a purinergic activator was chosen to avoid a confounding influence on ATP measurements and to determine if P2Y agonists had a significant role. This finding is supported by the observation that P2Y receptor activation is the primary pathway that contributes to the rise of intracellular Ca^2+^ in native urothelial cells in response to ATP stimulation (53). UTP-induced ATP release was also consistently observed in cultured rat urothelial cells (13). Thus, P2Y-mediated ATP release represents an important autocrine and/or paracrine feedback mechanism to amplify ATP-induced ATP release. Although UTP (ATP)-induced ATP release is mostly relevant to the urothelial cell layer, this mechanism may also exist in the suburothelial cell network, which amplifies the response in the functional syncytium of the urothelium/suburothelium.

An important role for muscarinic receptors in urothelial function has also been proposed (1, 2). Immunostaining has shown the expression of several subtypes of muscarinic receptors in the human and animal urothelium (10, 20, 57), which represent important therapeutic targets (10, 20, 57). The most recent functional experiments using native urothelial preparations (53) suggest that the phenotype of urothelial muscarinic receptor activation is distinct from purinergic receptors in several ways: *1*) muscarinic receptors do not participate in the generation of a Ca^2+^ rise in urothelial cells; *2*) these receptors have little influence on the ATP-evoked Ca^2+^ response; and *3*) they attenuate transurothelial membrane potential, unlike the positive effect of purinergic receptors. These observations suggest a different mode of action for muscarinic neurotransmitters in the urothelium. Here, we have shown that muscarinic receptor activation results in significant ATP release from intact urothelium specimens. Our finding from native urothelium tissue is corroborated by muscarinic agonist-triggered ATP release in cultured urothelial cells from the rat (26), and, more recently, from the human bladder (8). All these consistent observations demonstrate that muscarinic activation-induced ATP release is not only a fundamental biological phenomenon in experimental systems but, more importantly, has physiological relevance. As stretch-dependent ACh release from the urothelium is increased in aging bladders (2, 54), this may contribute to their enhanced ATP release by activating muscarinic receptors.

The receptor subtypes responsible for the muscarinic actions cannot be stated precisely as the receptor antagonists have imperfect subtype selectivity. However, it is likely that M_2_ receptors are involved in part, as methoctramine had an inhibitory effect and oxotremorine enhanced the release at concentrations that showed preferential selectivity to M_2_ receptors. This should provide specific selectivity over detrusor-active agents, where the active subtype is M_3_. This distinction is of importance in aging bladders, where M_2_ receptor expression remains unchanged, whereas M_3_ receptors are reduced (33), thus providing relatively greater M_2_ activity. Thus, the increased intrinsic ATP release, enhanced stretch-dependent ACh release, and preponderance of M_2_ activity may contribute to the urothelium-associated predisposing factors for overactivity in the aging bladder. A previous study (26) in cultured rat urothelial cells showed that M_1_, M_2_, and M_3_ receptors were all involved in ATP release. The differences between cultured urothelial cells and the intact urothelial sheet suggest that subtype selection occurs in fully differentiated native tissue, whereas all mechanisms are present in proliferating phenotypes of cultured cells. A recent study (55) has shown that muscarinic receptors might also be involved in stretch-induced ATP release in the urothelium under specific stretch protocols; however, M_3_ instead of M_2_ receptors seemed to be predominant.

Both purinergic and cholinergic subtypes involved in urothelial function in our study are distinct from their smooth muscle counterparts. In the urothelium, P2Y and M_2_ receptors are involved in activation, whereas P2X and M_3_ receptors are involved in activation in detrusor smooth muscle. This suggests urothelium-specific regulation. Furthermore, the ability of muscarinic agonists to modulate ATP release confers a mode of interaction between cholinergic and purinergic systems, and this, together with the autocrine modulation to trigger further ATP release by purinergic activation, constitutes complex regulatory mechanism to coordinate and amplify signal transduction in the urothelium.

### Mucosal Contraction

A further finding from this study is that a proportion of mucosal sheets were capable of generating small spontaneous contractions and, in other specimens, muscarinic and, to a lesser extent, purinergic activation generated contractile responses. The nature of this spontaneous contractile activity in guinea pig mucosa, which was also seen in human specimens (see below) differs from the spontaneous activity in the porcine urothelium/lamina propria, which involves muscarinic M_3_ receptors (36). The spontaneous contractions observed in our experiments were not sensitive to atropine (data not shown), and, also, mucosal contraction could be induced by other agonists in addition to muscarinic activators. The nature of the contraction is not fully understood. These contractions are unlikely to solely originate from the muscularis mucosae, as it represented, at most, 8% of the total tissue mass (N. Kushida and C. H. Fry, unpublished observations). Another possibility is a contribution from suburothelial myofibroblasts, which also contain contractile proteins, as similar cells in the rat intestine generate contractions via a P2Y2 receptor-dependent pathway (38). This ability of the mucosa to contract as a functional unit has important physiological implications as they could generate local stretch to sensory nerves as well as the urothelium itself and thus augment ATP release. This regulation of urothelial function by contraction of the mucosa represents a further feedback control over ATP release.

### Pathways for ATP Release

While ATP release has been demonstrated in a wide range of cell types, the pathways for its release in many native tissues are poorly understood, in particular for receptor-mediated ATP release as opposed to stretch-related release; the majority of the evidence has been gathered from cultured cells, where lytic action may lead to high levels of ATP release (28, 32, 40). ATP release from tissue specimens is multifactorial (29, 44, 49) and is likely to be tissue specific (40). This study demonstrated two physiological features in the bladder: *1*) mucosal contractions were frequently accompanied by an increase of ATP release and *2*) agonists such as UTP evoked both a rise of intracellular Ca^2+^ and ATP release. These observations suggest that mucosal contractions and an increase of urothelial intracellular Ca^2+^ rise are important determinants for ATP release.

Muscle contraction may lead to ATP release, and this release is via mechano-sensitive channels, as opposed to vesicular formation or trafficking (30), as observed in cultured urothelial cells (5). The ability of the mucosa to contract and the fact that urothelial cells express functional stretch-sensitive channels (53) suggest that a component of ATP release is via stretch-sensitive channels. This is further supported by urothelial ATP release under external mechanical stretch, as shown in this study. This mode of action may contribute to carbachol-induced ATP release and, to a lesser extent, UTP-induced ATP release in urothelial tissue. However, it is clear that other Ca^2+^-dependent and Ca^2+^-independent pathways must exist, as activation of P2Y receptors by UTP generated lower levels of contraction but significant increases in intracellular Ca^2+^ and ATP release and muscarinic agonists release ATP but do not generate a rise in intracellular Ca^2+^.

The pathways underlying intrinsic basal ATP release in the urothelium are mainly attributable to vesicular transport or exocytosis and, to a smaller extent, to pannexin hemichannel conductive efflux, as evidenced by a significant suppressant effect of the vesicle trafficking inhibitor brefeldin A and a moderate attenuation by the gap junction blocker carbenoxolone as well as the additive effect in the presence of both agents. Different cellular pathways are also responsible for UTP-mediated Ca^2+^-dependent ATP release and carbachol-mediated Ca^2+^-independent ATP release. UTP-mediated ATP release is due to intracellular Ca^2+^ release rather than extracellular Ca^2+^ entry, a feature of P2Y receptor signaling. This finding is supported by the inhibitory effect of the cell-permeable Ca^2+^ chelator BAPTA-AM and the lack of effect of removal of extracellular Ca^2+^. In contrast, carbachol-induced ATP release is cAMP dependent, as evidenced by the loss of its excitatory effect in the presence of the adenylyl cyclase inhibitor SQ-22536, consistent with M_2_ receptor coupling. These observations highlight two distinct cellular mechanisms underlying purinergic and muscarinic receptor-mediated ATP releases in the urothelium in addition to the common pathways of ATP release in this tissue. This is further distinguished by their dependence on vesicular trafficking with carbachol-evoked ATP release suppressed by brefeldin A, whereas UTP-triggered ATP release was largely unaffected. It is interesting to note that intrinsic basal ATP release from the urothelium is likely to be governed by different mechanisms, as neither BAPTA-AM nor SQ-22536 produced an effect.

### Paracrine Action of ATP Release

Using whole tissue mounts, this study has demonstrated a paracrine action of urothelial ATP release with detrusor smooth muscle contraction providing an effective biosensor. With an intact urothelium, spontaneous and low-concentration agonist-induced contractions were seen. These would normally be absent when the mucosa was removed. These contractions were attenuated by ABMA, which desensitizes P2X receptors that are present in smooth muscle, but has no effect on urothelial cells. This suggests that endogenously released ATP as well as that triggered by agonists is sufficient to generate a paracrine effect on smooth muscle. This finding has profound implications: it means that urothelial ATP release can provide a chemical signal to affect deep smooth muscle and is sufficient to stimulate other structures in the urothelium and suburothelium compartments, such as sensory nerve endings and interstitial cells, which may contribute to sensory modulation. The net ATP in the extracellular space for biological effect is, however, not only governed by ATP release from cells but also regulated by local factors, in particular, breakdown by ectonucleotidases (56). The autocrine and paracrine effect can occur in many cell types in the bladder wall expressing purinoceptors.

### Relevance to Human Conditions

The important findings from this study were also tested on human bladder mucosal biopsies. Our results showed that intact human urothelial patches could also actively release ATP, with spontaneous surges of such release. Likewise, purinergic activators as well as muscarinic agonists could evoke ATP release, and the effect was at least as large as that in guinea pig tissue. Low-amplitude contractile activity was also demonstrated in human preparations. The mucosal contraction could additionally be generated by muscarinic agonists and was again associated with urothelial ATP release. Therefore, the major findings from guinea pig experiments were consistently demonstrated in human urothelial tissue. These findings give credence to our observations from the guinea pig model and suggest that neurotransmitter-mediated ATP release and its subsequent paracrine action are important elements for human urothelial function. The influence of age on basal release from human preparations remains to be established as the age range of patients in this study was relatively limited but is of importance in view of the greater prevalence of sensory urgency in older people (15).

### Limitations of the Study

The continuous perfusion system used in this study permits measurement of dynamic changes of ATP release from tissue specimens and minimizes the effect of accumulated metabolites. It is possible that ATP released from the tissue was underestimated, and extrapolation to absolute levels of ATP released should be made with caution; the results should be considered in conjunction with those obtained from a static system such as cell culture to gain a more complete picture of ATP release in the urothelium.

### Conclusions

A native urothelium/suburothelial tissue specimen behaves as a functional unit with the following characteristics: *1*) the tissue actively releases ATP, a process that is altered during aging; *2*) purinergic and muscarinic neurotransmitters exert significant control over ATP release from the urothelium, probably via P2Y and M_2_ receptors, respectively, by distinct mechanisms; *3*) the mucosa has small intrinsic contractile activity that can be modulated by muscarinic agonists; *4*) mucosal contractile activity and intracellular Ca^2+^ rises are associated with ATP release; and *5*) released ATP from the urothelium either spontaneously or by muscarinic activation produces a paracrine effect on the underlying structure. These findings provide new information on the key physiological pathways in urothelial signaling and also warrant further translational exploration.

## GRANTS

This work was supported by the Biotechnology and Biological Sciences Research Council, the Wellcome Trust, Age UK, the Pfizer Overactive Bladder programme, and EU FP7 INComb for financial support.

## DISCLOSURES

No conflicts of interest, financial or otherwise, are declared by the author(s).

## AUTHOR CONTRIBUTIONS

Author contributions: G.S., C.H.F., B.M., and C.W. conception and design of research; G.S., M.R., R.W., and C.W. performed experiments; G.S., R.W., and C.W. analyzed data; G.S., C.H.F., R.W., and C.W. interpreted results of experiments; G.S. and C.W. drafted manuscript; G.S., C.H.F., B.M., R.W., and C.W. edited and revised manuscript; G.S., C.H.F., B.M., M.R., R.W., and C.W. approved final version of manuscript; M.R. and C.W. prepared figures.
